# Knowledge of cervical cancer screening, barriers to awareness, and the role of technological interventions in improving screening uptake: a scoping review protocol

**DOI:** 10.3389/frhs.2026.1857077

**Published:** 2026-08-06

**Authors:** Slindile Zondi, Mabina Mogale, Elizabeth Khalongo

**Affiliations:** Department of Public Health, Sefako Makgatho Health Sciences University Faculty of Health Sciences, Pretoria, South Africa

**Keywords:** cervical cancer screening, digital health interventions, health awareness, scoping review, screening uptake

## Abstract

**Background:**

Cervical cancer remains a major public health concern despite being largely preventable through early detection, regular screening, timely diagnosis, and effective treatment of precancerous lesions. Globally, cervical cancer continues to be one of the leading causes of cancer-related morbidity and mortality among women, particularly in low- and middle-income countries where access to preventive services remains limited. Although screening guidelines from the World Health Organization (WHO) and the South African Department of Health (DOH) are widely available, inadequate knowledge and poor dissemination of information to women continue to hinder participation in screening programmes. In South Africa, studies have highlighted inconsistencies and ineffective health education related to cervical cancer screening, diagnosis, and treatment, resulting in low screening uptake and delayed healthcare-seeking behaviour. The lack of structured cervical cancer education within public healthcare settings contributes to delayed diagnosis, increased mortality, higher healthcare costs, and psychological distress among affected women. In response, technological interventions such as reminder systems, callback mechanisms, mass media campaigns, mobile health (mHealth) programmes, and digital health strategies have been recommended to improve awareness, knowledge, and screening uptake among women.

**Methods and analysis:**

The development of this protocol follows the methodological framework proposed by Arksey and O'Malley for conducting scoping reviews, with enhancements recommended by Levac et al. and the Joanna Briggs Institute. The review selection process will be reported using the Preferred Reporting Items for Systematic Reviews and Meta-Analyses Extension for Scoping Reviews (PRISMA-ScR) to ensure methodological transparency and reproducibility. Rayyan will be used to facilitate the screening process and identify duplicate records during study selection. The population of interest will include women eligible for cervical cancer screening irrespective of age, with particular attention to adolescents, older women, rural residents, underserved populations, and healthcare providers where relevant. The review will focus on knowledge and awareness of cervical cancer, barriers to screening participation, and technological interventions including mHealth, SMS-based systems, telemedicine, and digital health platforms aimed at improving screening uptake and health education. Searches will be conducted on the Web of Science, PubMed, Google Scholar, and Scopus to identify relevant literature. A two-stage screening process involving title/abstract screening followed by full-text review will be used to determine study eligibility, and data will be extracted using a standardized data-charting form.

**Clinical Trial Registration:**

https://osf.io/4ms7w.

## 1 Introduction

Cervical cancer remains a significant global public health concern and a leading cause of morbidity and mortality among women, despite being one of the most preventable cancers through effective screening and early intervention (1, 2). Substantial disparities in incidence, mortality, and screening coverage persist across and within countries, with women in low- and middle-income countries (LMICs) and marginalized populations in high-income settings being disproportionately affected (1, 3). These inequities are largely driven by inadequate knowledge, limited awareness of screening services, and persistent structural and health system barriers that hinder access to timely screening and care (4, 5).

Knowledge and awareness of cervical cancer screening including understanding of risk factors, eligibility criteria, recommended screening intervals, and available screening modalities are key determinants of screening uptake (6). However, evidence consistently shows that many women have low to moderate levels of awareness, often compounded by misconceptions, fear, stigma, and sociocultural beliefs that negatively influence health-seeking behavior (7, 8). In addition, structural barriers such as limited access to healthcare services, financial constraints, long waiting times, and poor communication with healthcare providers further reduce participation in screening programmes (9, 10).

In response to these challenges, technological and digital health interventions have emerged as promising strategies to improve cervical cancer screening awareness and uptake. These include mobile health (mHealth) applications, short message service (SMS) reminders, telemedicine platforms, artificial intelligence-driven tools, and point-of-care innovations such as HPV self-sampling and visual inspection technologies (11, 12). These interventions offer scalable and potentially cost-effective approaches to addressing both informational and structural barriers to screening (13, 14).

Despite the expanding body of literature in this field, the evidence base remains fragmented and inconsistently synthesized. Existing studies often focus on isolated components, such as knowledge levels, individual barriers, or specific intervention types, without adequately integrating how these factors interact across different populations and health system contexts (15). Furthermore, prior reviews tend to be narrow in scope, concentrating on specific technologies, populations, or geographic regions, rather than providing a comprehensive mapping of knowledge, barriers, and technological interventions within a unified framework.

Additionally, heterogeneity in study designs, definitions, and outcome measures further limits comparability across studies, making it difficult to draw consolidated conclusions to inform policy and practice (1). Importantly, the gap in the literature is not the absence of research, but rather the lack of a systematically mapped and integrated synthesis that brings together evidence on knowledge, barriers, and technological interventions in cervical cancer screening across diverse settings.

Therefore, a scoping review is warranted to comprehensively map the available evidence on cervical cancer screening knowledge, barriers, and technological interventions. This approach is consistent with established scoping review methodology designed to map key concepts and identify gaps in evidence (16, 17). This review will provide an overview of existing literature, identify key gaps, and highlight opportunities for future research and implementation strategies aimed at improving cervical cancer screening uptake globally.

## 2 Methods

**Figure 1:**
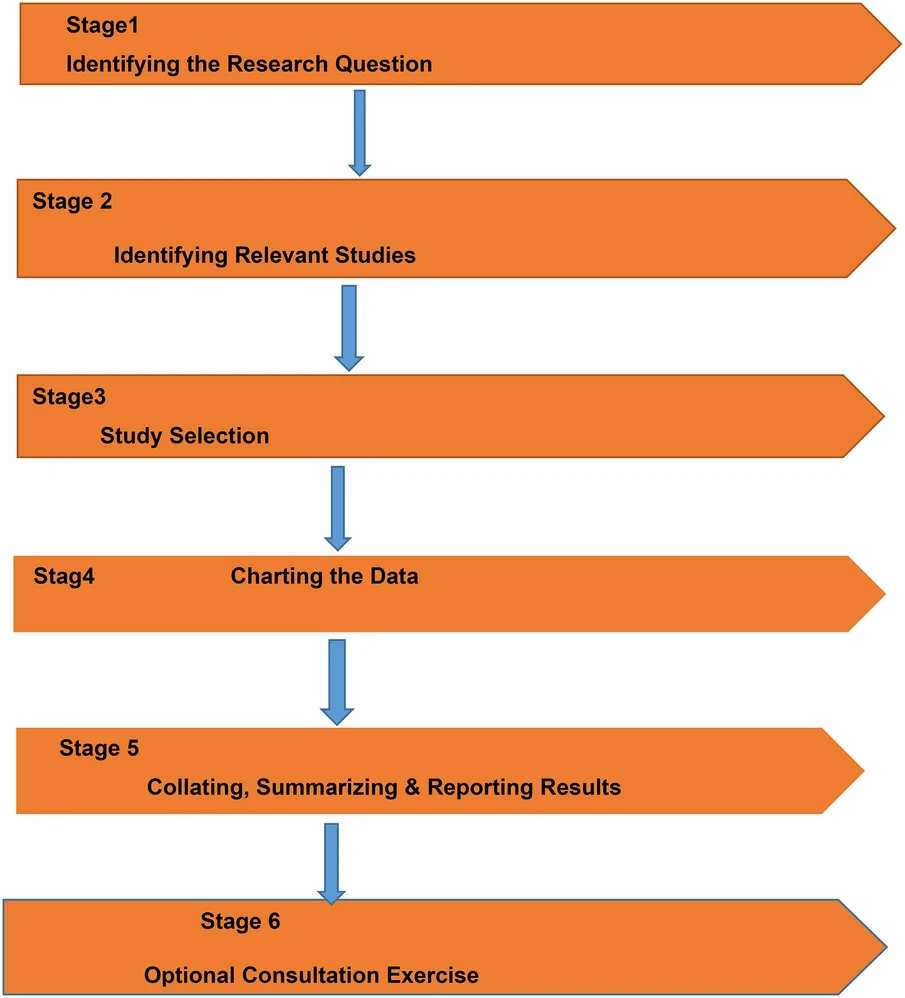
Arksey & O’Malley six-stage scoping review framework.

This scoping review will be conducted in accordance with the Joanna Briggs Institute (JBI) methodology for scoping reviews, informed by the foundational framework proposed by Arksey and O’Malley, and refined by (18). The reporting of this review will follow the PRISMA-ScR (Preferred Reporting Items for Systematic Reviews and Meta-Analyses Extension for Scoping Reviews) guidelines. Figure 1 shows six-step framework for scoping reviews of Arksey and O’Malley. The Prisma-ScR flow diagram will be used to illustrate the process of the study selection. The population of interest will include women eligible for cervical cancer screening, irrespective of age, with particular attention to key sub-populations such as adolescents, older women, rural residents, and underserved or marginalized groups as well as healthcare. The review will focus on three interrelated concepts: (i) knowledge and awareness of cervical cancer and cervical cancer screening; (ii) barriers to cervical cancer knowledge and screening participation at the individual, sociocultural, and health system levels; and (iii) technological and technical interventions aimed at improving awareness and screening uptake, including mHealth and SMS-based interventions, digital health and telemedicine platforms. The context of the review will encompass all geographical regions and settings, including clinical, community-based, and digital health environments, across high-, middle-, and low-income countries. Searches will be conducted on the Web of science, PubMed, Google Scholar, and Scopus. A 2-stage screening process will determine study eligibility, and data will be extracted using a standardized form.

The search strategy will involve systematic searches of articles in major electronic databases, including Web of science, PubMed and Scopus as well as other relevant sources including Google Scholar (19). The registration of this protocol for the scoping review has been accepted by the Open Science Framework and can be accessed at https://osf.io/4ms7w. Changes to this protocol will be reported in the results section.

### 2.1 Stage 1: develop research questions

The PCC framework will be applied to formulate research questions with a focus on the following key elements: women (Population), (i) knowledge and awareness of cervical cancer and cervical cancer screening; (ii) barriers influencing screening participation at the individual, sociocultural, and health system levels; and (iii) technological and technical interventions aimed at improving awareness and uptake, including mHealth, SMS-based interventions, digital health, and telemedicine platforms (Concept) encompasses all geographical regions and settings, including clinical, community-based, and digital health environments across high-, middle-, and low-income countries (Context). Therefore, the following research questions will guide this scoping review.

Review Questions

1. What gaps in knowledge and awareness of cervical cancer and cervical cancer screening among eligible women have been identified in the literature?
2. What barriers influence women’s knowledge of cervical cancer and their participation in cervical cancer screening programmes?
3. How do technological and digital health interventions address identified barriers and knowledge gaps related to cervical cancer screening uptake?
4. What evidence exists on the effectiveness of technological interventions in improving awareness and participation in cervical cancer screening?
5. What gaps in the existing literature warrant further research, innovation, and policy development in cervical cancer screening education and uptake?

### 2.2 Stage 2: identify relevant literature

A comprehensive and iterative search strategy was developed in consultation with an academic librarian at Sefako Makgatho Health Science University in South Africa. The following databases will be searched: PubMed, Scopus and Web of Science. Grey literature sources will include Google scholars. Relevant keywords will be used to identify pertinent papers through Boolean operator searches, such as “AND/OR”. The review will only consider literature published in English. Keywords will include the following components:

Knowledge & Awareness
  (“cervical cancer” OR “cervical neoplasm” OR “cervical screening” OR “cervical cancer screening” OR “Pap smear” OR “HPV screening”) AND (knowledge OR awareness OR perception OR understanding OR literacy) AND (“eligible women” OR women OR female* OR “women of screening age” OR “reproductive age women”) *
Barriers to Screening
  (“cervical cancer screening” OR “cervical screening” OR “Pap smear” OR “HPV screening”) AND (barrier OR challenge OR obstacle* OR determinant* OR factor* OR perception* OR attitude*) AND (knowledge OR awareness) AND (participation OR uptake OR utilization OR attendance OR adherence) AND (women OR female*) *
Digital health
  “cervical cancer screening” OR “cervical screening” OR “Pap smear” OR “HPV screening”) AND (“digital health” OR technology OR mHealth OR eHealth OR telehealth OR chatbot OR “conversational agent” OR “mobile app*” OR “SMS reminder*”) AND (barrier* OR challenge* OR obstacle* OR “knowledge gap*” OR awareness OR education) AND (uptake OR participation OR utilization OR attendance) *
“Cervical cancer screening” OR “cervical screening”) AND (“digital intervention” OR “technology-based intervention” OR chatbot* OR mHealth OR eHealth OR telemedicine) AND (effectiveness OR efficacy OR impact OR outcome* OR evaluation) AND (awareness OR knowledge OR participation OR uptake OR attendance)
(“cervical cancer screening” OR “cervical screening”) AND (“digital health” OR technology OR chatbot OR “AI intervention” OR mHealth) AND (“research gap*” OR “knowledge gap*” OR limitation* OR “future research” OR innovation OR policy OR guideline* OR recommendation*)

### 2.3 Stage 3: study selection

All retrieved references will be imported into a reference management software program for the identification and removal of duplicate records before being uploaded into Rayyan to facilitate the screening process. The study selection process will be guided by the eligibility criteria developed using the Population, Concept, and Context (PCC) framework. Two independent reviewers will screen all identified studies to ensure consistency and minimise selection bias. The screening process will be conducted in two steps: first, titles and abstracts will be reviewed to identify potentially relevant studies; second, the full texts of eligible studies will be assessed against the predefined inclusion and exclusion criteria. Any disagreements between reviewers will be resolved through discussion and consensus, and where agreement cannot be reached, a third reviewer will be consulted. Reasons for excluding studies at the full-text screening stage will be documented and reported. The study selection process will be presented using the Preferred Reporting Items for Systematic Reviews and Meta-Analyses Extension for Scoping Reviews (PRISMA-ScR) flow diagram to ensure transparency and reproducibility. This process will be conducted in accordance with the scoping review methodological framework developed by Arksey and O'Malley and further refined by Levac et al., which provides a rigorous and systematic approach to identifying, selecting, and mapping the available evidence while highlighting existing research gaps (16, 20).

The criteria for eligibility will be determined using the PCC framework, which is outlined in Table 1.

**Table 1:** Population–Concept–Context (PCC) framework used to define the eligibility criteria for the scoping review.

PCC element	Description
Population	Women eligible for cervical cancer screening, irrespective of age, including adolescents, older women, rural populations, underserved and marginalized groups, and healthcare providers where relevant
Concept	(i) Knowledge and awareness of cervical cancer and cervical cancer screening; (ii) barriers to screening participation at individual, sociocultural, and health system levels; (iii) technological and digital health interventions such as mHealth, SMS reminders, telemedicine, and digital platforms
context	All geographical settings including clinical, community-based, and digital health environments across high-, middle-, and low-income countries

### 2.4 Stage 4: charting the data

Eligible studies will undergo data charting using a standardized Microsoft Excel-based charting form that aligns with PRISMA-ScR guidelines. This form will be utilized to extract essential information, including the author, year, study setting, study population, level of awareness, identified barriers, digital interventions used, and key findings. The charting process will be iterative, allowing for refinement of the tool as new themes emerge during the review.

### 2.5 Stage 5: summarize results

The data synthesis and analysis will utilize a compelling combination of descriptive and thematic synthesis to effectively showcase the evidence gathered from the included studies. In alignment with PRISMA-ScR guidelines, this synthesis aims not only to map and summarize the evidence pertinent to the research questions but also to emphasize the critical findings that can drive impactful changes in cervical cancer awareness and screening. Initially, we will organize the data into comprehensive summary tables that provide a clear overview of study characteristics, including authorship, publication year, setting, design, population details, and crucial insights on cervical cancer knowledge, awareness, barriers to screening, and the efficacy of digital health interventions. A descriptive numerical analysis will illuminate the distribution of studies by year, location, design, and population, revealing trends that warrant attention. Following this, we will conduct a thematic analysis to uncover significant patterns and themes across studies, such as varying levels of knowledge and awareness, socio-cultural and health system barriers, common misconceptions, and the potential of conversational agents and digital tools to facilitate increased screening uptake. Finally, we aim to present our findings in both narrative and tabular formats, effectively mapping existing evidence while highlighting knowledge gaps. Our recommendations on utilizing conversational agents to enhance cervical cancer screening awareness and participation have the potential to make a tangible difference in public health outcomes.

### 2.6 Stage 6: consult with expert

Consultation with key stakeholders has been incorporated throughout the development of this scoping review protocol. Academic supervisors, specialised librarians, and an experienced language editor have already contributed to the refinement of the review question, search strategy, and overall protocol development. Their expertise has helped to strengthen the methodological rigour and clarity of the review process. Ongoing consultation with these stakeholders will continue, where necessary, during the review to ensure methodological consistency and comprehensiveness. The findings of the scoping review will be disseminated through academic publications, conference presentations, and reports to relevant stakeholders to enhance knowledge translation and inform future research, policy, and practice.

## 3 Ethical considerations

As no human participants will be involved in this study, ethical approval will not be required. The scoping review protocol will be registered with the Open Science Framework (OSF) and will be accessible at: Open Science Framework Registration https://osf.io/4ms7w.

## 4 Discussion and dissemination

The discussion of findings will critically interpret the synthesized evidence in relation to the study's objectives and research questions. It will underscore significant patterns in cervical cancer knowledge and awareness, pinpoint major barriers to screening participation, and examine the impactful role of digital health interventions, particularly conversational agents, in bridging knowledge gaps. Furthermore, this discussion will compare findings across various contexts, highlighting consistencies, variations, and gaps in the existing literature. These valuable insights will drive informed recommendations aimed at enhancing cervical cancer screening awareness and promoting the effective integration of conversational agents in healthcare settings. To ensure the results reach the appropriate academic, clinical, and policy audiences, we will strategically disseminate our findings. They will be presented in a master's dissertation and may also be submitted for publication in a peer-reviewed journal. Additionally, we will share our results at academic conferences, seminars, and through engaging with stakeholders, including healthcare professionals and public health policymakers. A brief and impactful summary of the findings will be tailored for non-academic audiences to foster community awareness and guide future digital health interventions designed to boost cervical cancer screening uptake.

## References

[1] World Health Organization (WHO). Cervical Cancer Fact Sheet. Geneva: World Health Organization (2024).

[2] Arbyn M Weiderpass E Bruni L de Sanjosé S Saraiya M Ferlay J. Estimates of incidence and mortality of cervical cancer in 2018: a worldwide analysis. Lancet Glob Health. (2020) 8(2):e191–203. 10.1016/S2214-109X(19)30482-6 31812369 PMC7025157

[3] Sung H Ferlay J Siegel RL Laversanne M Soerjomataram I Jemal A. Global cancer statistics 2020: GLOBOCAN estimates of incidence and mortality worldwide for 36 cancers in 185 countries. CA Cancer J Clin. (2021) 71(3):209–49. 10.3322/caac.21660 33538338

[4] Marlow LAV Waller J Wardle J. Barriers to cervical cancer screening among ethnic minority women. J Med Screen. (2019) 22(3):153–9.

[5] Crawford A Benard V King J Thomas CC. Understanding barriers to cervical cancer screening in women with access to care, behavioral risk factor surveillance system, 2014. Prev Chronic Dis. (2016) 13:E154. 10.5888/pcd13.160225 27831682 PMC5109933

[6] Musa J Achenbach CJ O’Dwyer LC Evans CT McHugh M Hou L. Effect of cervical cancer education and provider recommendation on screening rates: a systematic review. PLoS One. (2017) 12(9):e0183924. 28873092 10.1371/journal.pone.0183924 PMC5584806

[7] Lim JNW Ojo AA. Barriers to utilisation of cervical cancer screening in sub-Saharan Africa: a systematic review. Eur J Cancer Care (Engl). (2017) 26(1):e12444. 10.1111/ecc.12444 26853214

[8] Ndejjo R Mukama T Musabyimana A Musoke D. Uptake of cervical cancer screening and associated factors among women in Uganda: a systematic review. PLOS One. 12(5):e0176711. 10.1371/journal.pone.017671 26894270 PMC4760951

[9] Durowade KA Osagbemi GK Salaudeen AG Babatunde OA Bolarinwa OA Ajayi PO. Knowledge, attitude and practice of cervical cancer screening among female students in a tertiary institution in Nigeria. Int J Med Biomed Res. (2012) 1(3): 216–27.

[10] Rashid S Labani S Das BC. Knowledge, awareness and barriers to cervical cancer screening among women: a systematic review. Clin Epidemiol Glob Health. (2022) 15:101010.

[11] Vodopivec-Jamsek V de Jongh T Gurol-Urganci I Atun R Car J. Mobile phone messaging for preventive health care. Cochrane Database Syst Rev. (2012) 12:CD007457. 10.1002/14651858.CD007457.pub2 23235643 PMC6486007

[12] Ndejjo R Mukama T Musinguzi G Halage AA Ssempebwa JC Musoke D. (2017). Women's intention to screen and willingness to vaccinate their daughters against cervical cancer: A cross-sectional study in eastern Uganda. BMC Public Health. 17(1):255. 28288614 10.1186/s12889-017-4180-4 PMC5348782

[13] Arrossi S Paolino M Laudi R Campanera A Wang D Sankaranarayanan R. Programmatic HPV testing in cervical cancer prevention in Latin America. Salud Pública de México. (2015) 57(6):504–12.26679313

[14] Yeh PT Kennedy CE de Vuyst H Narasimhan M. Self-sampling for human papillomavirus (HPV) testing: a systematic review and meta-analysis. BMJ Glob Health. (2019) 4(3):e001351. 10.1136/bmjgh-2018-001351 31179035 PMC6529022

[15] Finocchario-Kessler S Wexler C Maloba M Mabachi N Ndikum-Moffor F Bukusi E. Cervical cancer prevention and treatment research in low-resource settings: a systematic review. BMC Women’s Health. (2016) 16(29):1–13. 27259656 10.1186/s12905-016-0306-6 PMC4893293

[16] Arksey H O'Malley L. Scoping studies: towards a methodological framework. Int J Soc Res Methodol. (2005) 8(1):19–32. 10.1080/1364557032000119616

[17] Peters MDJ Marnie C Tricco AC Pollock D Munn Z Alexander L. Updated methodological guidance for the conduct of scoping reviews. JBI Evidence Synthesis. (2020) 18(10):2119–26. 10.11124/JBIES-20-00167 33038124

[18] Levac L Ronis S Cowper-Smith Y Vaccarino O. A scoping review: the utility of participatory research approaches in psychology. J Community Psychol. (2019) 47(8):1865–92. 10.1002/jcop.22231 31441516 PMC6852237

[19] Sokhulu LH Nzimande N Makumane MA. Postgraduate students’ experiences of using EndNote in research studies. Soc Sci Educ Res Rev. (2024) 11(1):227–37. 10.5281/zenodo.15258173

[20] Levac D Colquhoun H O’Brien KK. Scoping studies: advancing the methodology. Implement Sci. (2010) 5(69):1–9. 10.1186/1748-5908-5-69 20854677 PMC2954944

